# Japanese traditional herbal medicine reduces use of pregabalin and opioids for pain in patients with lumbar spinal canal stenosis: a retrospective cohort study

**DOI:** 10.1186/s40981-017-0130-5

**Published:** 2017-10-27

**Authors:** Mitsuhiko Oohata, Yuko Aoki, Michiko Miyata, Hiroki Mizobe, Kenji S. Suzuki

**Affiliations:** Department of Anesthesiology, School of Medicine, Iwate Medical University, 19-1 Uchimaru, Morioka-shi, Iwate, 020-8505 Japan

**Keywords:** Japanese traditional herbal medicine, Lumbar spinal canal stenosis, Pain management, Kampo, Oriental medicine

## Abstract

**Background:**

There has been an increase in the number of Japanese patients with lumbar spinal canal stenosis (LSCS) who complain of chronic pain or motor disturbance in the lower back or extremities. These patients are often treated with anti-convulsive drugs, opioids, antidepressants, acetaminophen, or nonsteroidal anti-inflammatory drugs, all of which can cause side effects. For this reason, Japanese traditional herbal medicine (Kampo) is of interest, because it produces fewer adverse reactions. The aim of this retrospective cohort study was to analyze the effects of Kampo in patients with LSCS.

**Findings:**

A total of 151 patients with LSCS were divided into two groups based on treatment with (*n* = 111, group K) and without (*n* = 40, group N) Kampo. Use of pregabalin and opioids decreased significantly in group K (*p* < 0.001). The hazard ratio for opioid discontinuation was 0.220 (*p* = 0.004) for group N vs. group K, while that for pregabalin and antidepressants discontinuation were 0.589 (*p* = 0.202) and 0.509 (*p* = 0.377), respectively. The mean duration of hospital visits and treatment did not differ between the groups, but the number of dropouts was significantly higher in group N (*p* < 0.0001). The hazard ratio for patient dropout was 4.118 (*p* = 0.001) for group N vs. group K.

**Conclusions:**

Kampo led to discontinuation of opioid use for pain in patients with LSCS, and patients who were treated with Kampo were more likely to continue treatment.

## Findings

### Introduction

The symptoms of lumbar spinal canal stenosis (LSCS), such as low back pain, lower limb pain, and intermittent claudication, often develop into chronic conditions. The increasing number of LSCS patients is a social issue because of the resulting decrease in labor productivity, reduction in health expectancy, and increase in health care costs [1, 2]. Pain clinics in Japan use nerve blocks, medications, and physiotherapy as treatments, but these therapeutic procedures are not always effective, and the degree of patient satisfaction can be low [3, 4]. We commonly prescribe anti-convulsants such as pregabalin, opioids, and antidepressants, in addition to nonsteroidal anti-inflammatory drugs and acetaminophen, for LSCS patients who experience pain. However, all these medicines have side effects.

Japanese traditional herbal medicine (referred to as Kampo) is of interest to pain clinics because it produces fewer side effects [5, 6]. In our clinic, Kampo is often prescribed for patients with pain.

We often encounter patients with pain who cannot use adequate doses of Western medicines, e.g., opioids or anti-convulsive drugs, because of their side effects, but can obtain moderate or high levels of satisfaction from Kampo.

The aims of this study were to analyze the recent use of Kampo in our outpatient pain clinic and to evaluate the effects of Kampo on outcomes with respect to its usability for pain management in outpatients. In addition, according to the hypothesis that a combination therapy using Kampo alongside Western medicine may have value, we examined the treatment progress of LSCS patients who complained of chronic low back pain, the most common condition in the outpatient department of our pain clinic in a retrospective cohort.

### Patients and methods

The study was approved by the ethics committee of Iwate Medical University Graduate School of Medicine (No H29-56). Clinical data for patients with LSCS admitted for ambulatory care between January 2015 and December 2016 were obtained from electronic records. LSCS patients who continued to visit our hospital for over 1 month were included in this study. To exclude acute nociceptive pain, we excluded subjects whose treatment ended within 1 month. The diagnosis of LSCS was made on the basis of clinical symptoms, e.g., low back pain, numbness in a lower extremity, or intermittent claudication seen in magnetic resonance imaging (MRI) of the lumbar spine. Observation and diagnosis using MRI were performed by radiologists in our institution. Patients with LSCS who received treatment for other painful diseases were excluded. A total of 151 patients were enrolled and divided into two groups; patients in group K were prescribed Kampo (*n* = 111), while those in group N were not (*n* = 40). Data were collected on the following patient characteristics: therapeutic regimens involving nerve block or near-infrared light therapy, rehabilitation, and drug prescriptions. We also assessed periods of hospital attendance, and outcomes that involved either an end to therapy, continuation of therapy, or dropout during the observation period. These data were compared between the two groups.

SPSS version 22 (SPSS Inc., Chicago, IL, USA) was used for statistical analysis. The Shapiro-Wilk test was used to determine whether data were normally distributed. Continuous data were expressed as median, 25th and 75th percentiles, or as mean ± standard deviation. Categorical variables were expressed as numbers or as percentages of patients. Continuous variables were compared using a Mann-Whitney *U* test or an unpaired Student’s *t* test. Categorical data were assessed using a Chi-square test. In addition, we performed an event history analysis to evaluate the influence of several variables on event risks. The event risks during the observation period for each group were analyzed using a Cox proportional hazard model, in which the independent valuables were taken from the results of a Chi-square test with significance level *p* < 0.1. A *p* value < 0.05 was considered to be significant.

### Results

The characteristics of the 151 patients in the study are shown in Table 1. About 60% of the patients were referrals from the Department of Orthopedics in our hospital, while fewer than 10% of patients had no letter of introduction.

**Table 1:** Demographic profile and source of referral of the patients

Item	Group K (*n* = 111)	Group N (*n* = 40)	*p* value
Age (years)	73.3 ± 9.4	70.8 ± 9.5	0.16
Male/female	60/51	21/19	0.87
Height (cm)	158.4 ± 10.4	158.3 ± 8.8	0.95
Weight (kg)	58.0 ± 11.7	62.3 ± 11.6	0.06
Source			
Our hospital			
Orthopedics (%)	68	58	
Neurosurgery (%)	3	12	
Neurology (%)	3	2	
Others (%)	1	0	
Another institution			
Pain clinic (%)	5	10	
Others (%)	10	8	
No introduction (%)	9	8	

Values are mean ± SD or number of patients

The formulas for Kampo prescriptions are shown in Table 2. Gosha-jinki-gan and Hachimi-jiō-gan were prescribed for many patients. Side effects due to Kampo occurred in seven patients: abdominal pain with Gosha-jinki-gan and Keishi-ka-jyutubu-tō, diarrhea with Shakuyaku-kanzo-tō and Bushi-matsu, excessive sweating and chest pain with Keishi-ka-jyutsubu-tō, and pseudoaldosteronism with Shakuyaku-kanzo-tō.

**Table 2:** Details of prescribed Kampo medicines

Name of drugs	Number of patients
Gosha-jinki-gan	51
Hachimi-jiō-gan	50
Shakuyaku-kanzo-tō	31
Keishi-ka-jyutsubu-tō	26
Bushi-matsu	24
Sokei-kakketsu-tō	15
Tokishigyaku-ka-goshui-shokyo-tō	12
Yokukan-san	10
Gorei-san	10
Others	65

The details of the effects of Kampo on the reduction of use of other drugs are shown in Table 3. The number of patients who discontinued use of pregabalin was significantly larger in group K than in group N (*p* = 0.0012). Similarly, the use of opioids discontinued more often in group K (*p* = 0.0002). On the other hand, the use of antidepressants slightly decreased in group K (*p* = 0.063). The estimated hazard ratio for discontinuation of opioids was 0.220 (95% CI 0.078–0.615, *p* = 0.004) for group N vs. group K, while that for pregabalin and antidepressants were 0.589 (95% CI 0.261–1.328, *p* = 0.202), 0.509 (95% CI 0.114–2.227, *p* = 0.377), respectively. The number of patients who discontinued use of NSAIDs and/or acetaminophen was not different between the groups (*p* = 0.891).

**Table 3:** The number of patients who were prescribed each drug and were followed-up

		Pregabalin	Opioid	Antidepressant	NSAIDs/acetaminophen
Group K (*n* = 115)	Prescription	69/111 (62.2%)	74/111 (66.7%)	22/111 (19.8%)	54/111 (48.6%)
	Decrease	45/69*** (65.2%)	52/74*** (70.3%)	17/22** (77.3%)	7/54 (13.0%)
	Discont	36/69*** (52.2%)	41/74*** (55.4%)	16/22* (72.7%)	6/54 (11.1%)
Group N (*n* = 44)	Prescription	19/40 (47.5%)	28/40 (70.0%)	6/40 (15.0%)	20/40 (50.0%)
	Decrease	2/19 (10.5%)	9/28 (32.1%)	2/6 (33.3%)	2/20 (10.0%)
	Discont	2/19 (10.5%)	4/28 (14.3%)	2/6 (33.3%)	2/20 (10.0%)

*Decrease* decrease in the dose of drug prescribed, *Discont* discontinuation of the drug prescribed

*p < 0.1; **p < 0.05; ***p < 0.01 vs. group N

The duration of hospital visits and treatment and the outcomes are shown in Table 4. The number of patients who dropped out, defined as not revisiting the hospital for more than 6 months during treatment, was significantly higher in group N (*p* < 0.0001). We did not ignore these dropout patients in all of the analysis. The hazard ratio for patient dropout was 4.118 (95% CI 1.792–9.460, *p* = 0.001) for group N vs. group K.

**Table 4:** Duration of hospital visits and outcomes

Patients	Group K (*n* = 111)	Group N (*n* = 40)	*p* value
Ended treatment Visiting period (months)	49.5% 12.0 (4.5, 24.5)	35.0% 5.0 (3.3, 16.3)	0.113 0.172
Continued treatment Visiting period (months)	42.3% 35.0 (20.5, 54.0)	27.5% 41.0 (19.0, 69.5)	0.098 0.592
Dropped out Visiting period (months)	8.1%* 8.0 (4.0, 26.0)	37.5% 34.0 (16.5, 50.5)	< 0.0001 0.172

Values are percentages of patients (%) and median (interquartile range)

*p < 0.05 vs. group N

### Discussion

Japanese traditional herbal medicine (Kampo) is covered by national medical insurance and widely used for pain management [7, 8]. Kampo is thought to be beneficial in terms of a lower incidence of side effects compared to Western medicine. However, substantial knowledge and experience are required to use Kampo effectively because the theory of Oriental traditional medicine is complicated and difficult to understand. In addition, education in Oriental medicine has been limited in Japan, and currently, there is an insufficient number of practitioners. Furthermore, the preparations include multiple herbal medicines, which makes it difficult to obtain pharmacological evidence of therapeutic effects. This difficulty is increased because Kampo is administered based on the pattern of a patient’s condition, rather than on a diagnosis, and the periods required for efficacy differ based on the kinds of formulations [9–12].

In this study, we assessed the effects of Kampo on the clinical course of patients with LSCS who complained of low back pain. The incidence of side effects caused by Kampo formulas was only 6.3% (7/111 patients), while 62.5% of patients treated without Kampo had side effects such as constipation, nausea, sleepiness, eruption, or edema. Therefore, the combined administration of Kampo decreased side effects compared to the use of Western-style medicines alone. The Cox proportional hazards model analysis showed that the estimated hazard ratio for discontinuing opioids was 0.220 (95% CI 0.078–0.615, *p* = 0.004) for group N vs. group K, while that for pregabalin and antidepressants were 0.589 (95% CI 0.261–1.328, *p* = 0.202) and 0.509 (95% CI 0.114-2.227, *p* = 0.377), respectively. This means that the treatment with Kampo medicine led to significant discontinuation of opioids treatment but not of pregabalin and antidepressants treatment.

The most important finding in the study was that Kampo prescriptions led to discontinuation of Western medications, especially opioids. In addition, patients who were prescribed Kampo continued to undergo treatment for longer periods.

This study has some inherent limitations. First, the retrospective design, small number of patients, and large difference in numbers in the two groups raise concerns about statistical validity. Second, the patients were divided into groups based on whether their prescriptions included Kampo, but we do not know whether this classification method was adequate. Third, we did not analyze the symptoms using an Oriental medical approach, and the evidence level for use of Kampo is still relatively low. Further analysis is therefore required, in a prospective trial of Kampo, to validate the findings in this study.

## Abbreviations

LSCS: Lumbar spinal canal stenosis
